# Recent advances in the improvement of cyanobacterial enzymes for bioalkane production

**DOI:** 10.1186/s12934-022-01981-4

**Published:** 2022-12-12

**Authors:** Yuuki Hayashi, Munehito Arai

**Affiliations:** 1 Department of Life Sciences, Graduate School of Arts and Sciences, The University of Tokyo, 3-8-1 Komaba, Meguro, Tokyo 153-8902 Japan; 2 Environmental Science Center, The University of Tokyo, 7-3-1 Hongo, Bunkyo, Tokyo 113-0033 Japan; 3 Department of Physics, Graduate School of Science, The University of Tokyo, 3-8-1 Komaba, Meguro, Tokyo 153-8902 Japan

**Keywords:** Hydrocarbons, Alkanes, Aldehydes, Biofuels, Cyanobacteria, Acyl-ACP reductase, Aldehyde deformylating oxygenase, Protein engineering, Protein–protein interaction

## Abstract

The use of biologically produced alkanes has attracted considerable attention as an alternative energy source to petroleum. In 2010, the alkane synthesis pathway in cyanobacteria was found to include two small globular proteins, acyl-(acyl carrier protein [ACP]) reductase (AAR) and aldehyde deformylating oxygenase (ADO). AAR produces fatty aldehydes from acyl-ACPs/CoAs, which are then converted by ADO to alkanes/alkenes equivalent to diesel oil. This discovery has paved the way for alkane production by genetically modified organisms. Since then, many studies have investigated the reactions catalyzed by AAR and ADO. In this review, we first summarize recent findings on structures and catalytic mechanisms of AAR and ADO. We then outline the mechanism by which AAR and ADO form a complex and efficiently transfer the insoluble aldehyde produced by AAR to ADO. Furthermore, we describe recent advances in protein engineering studies on AAR and ADO to improve the efficiency of alkane production in genetically engineered microorganisms such as *Escherichia coli* and cyanobacteria. Finally, the role of alkanes in cyanobacteria and future perspectives for bioalkane production using AAR and ADO are discussed. This review provides strategies for improving the production of bioalkanes using AAR and ADO in cyanobacteria for enabling the production of carbon–neutral fuels.

## Background

Hydrocarbons, specifically alkanes, which are produced by living organisms, may serve as an alternative energy source to fossil oils [1–6]. Alkanes are biologically produced by cyanobacteria, fungi, plants, and insects [7–10]. In particular, alkane biosynthesis using cyanobacteria is attractive because it enables the carbon–neutral production of alkanes using atmospheric carbon dioxide as a carbon source for photosynthesis, thereby contributing to the reduction of global warming caused by combustion of fossil fuel [11–15].

**Fig. 1:**
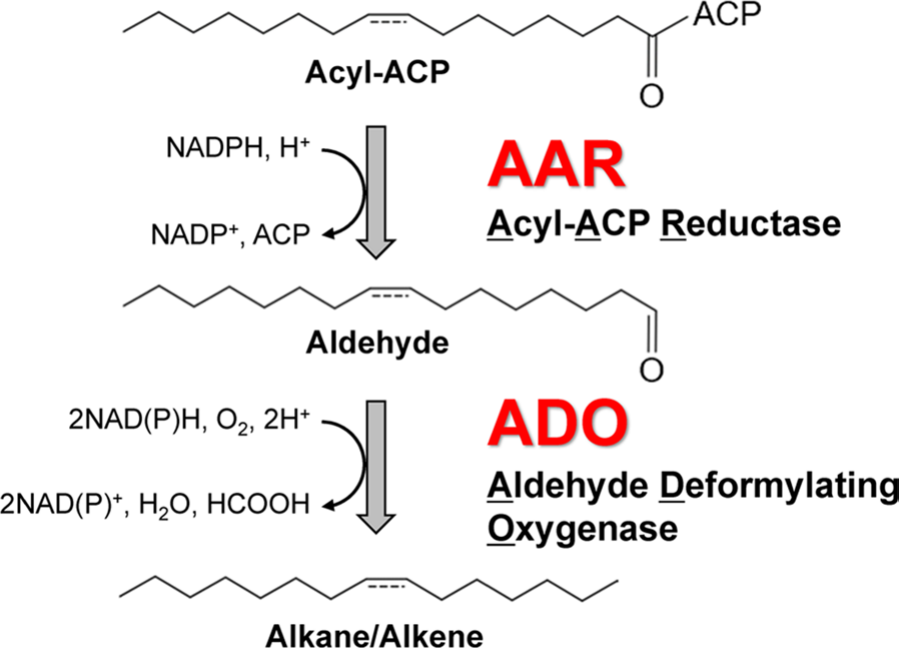
Cyanobacterial alkane biosynthesis pathway using AAR and ADO. AAR, acyl-(acyl carrier protein [ACP]) reductase; ADO, aldehyde deformylating oxygenase

From 1960s, cyanobacteria are known to produce alkanes [16, 17], and in 1980s, plants and algae have been suggested to contain aldehyde decarbonylase (AD), an enzyme that produces alkanes from aldehydes [18–21]. In 2010, Schirmer et al. identified two cyanobacterial enzymes for alkane biosynthesis: acyl-(acyl carrier protein [ACP]) reductase (AAR) and AD [22]. Cyanobacterial AD was later renamed as aldehyde deformylating oxygenase (ADO) because the byproduct of alkane synthesis from aldehydes was formate [23]. In a two-step reaction catalyzed by AAR and ADO, AAR first produces 16- or 18-carbon aldehydes (hexadecanal or octadecanal, respectively) from acyl-ACPs (or acyl-coenzyme As [CoAs]), which are intermediate products of fatty acid synthesis. Subsequently, ADO converts the aldehydes into 15- or 17-carbon alkanes (pentadecane or heptadecane, respectively) and formate (Fig. 1) [13, 22, 24–26]; ADO produces alkenes if the aldehyde substrates are monounsaturated. Notably, alkanes have been produced via heterologous expression of AAR and ADO in *Escherichia coli*, indicating that these enzymes are essential for alkane biosynthesis [13, 22]. Since acyl-ACPs are conserved in almost all organisms [27], alkane production can be achieved by co-expression of AAR and ADO in genetically engineered organisms.

Since our previous review on cyanobacterial enzymes for bioalkane production in 2018 [13], several studies have been conducted to characterize and improve AAR and ADO. In this review, we first summarize recent advances on the structure, function, and interaction of AAR and ADO. We then describe recent attempts made in protein engineering to improve hydrocarbon production using these enzymes.

### Structure and function of AAR and ADO

#### Structure and catalytic mechanism of AAR

**Fig. 2:**
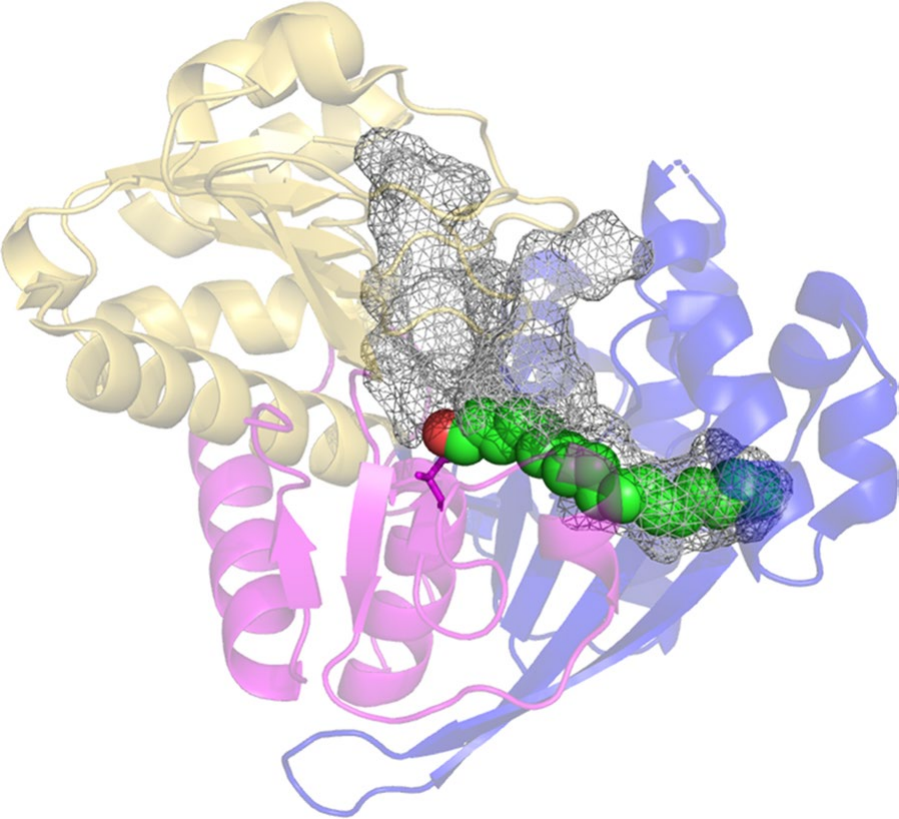
Crystal structure of AAR derived from *Synechococcus elongatus* PCC 7942 (PDB ID: 6JZU). AAR consists of three domains: N-terminal domain (blue), NADPH-binding domain (yellow), and C-terminal domain (magenta). The L-shaped hydrophobic tunnel is presented as a gray mesh. Octadecanal is shown in spheres. The red oxygen atom of the aldehyde is covalently attached to residue C294 (sticks) at the catalytic center. All figures of protein structures were generated using the PyMOL Molecular Graphics System, version 2.0.4 Schrödinger, LLC. AAR, acyl-(acyl carrier protein) reductase

AAR is a monomeric globular protein consisting of approximately 340 amino acid residues. The crystal structure of AAR was resolved in 2020 and revealed the presence of three domains: an N-terminal domain, a nicotinamide adenine dinucleotide phosphate (NADPH)-binding domain, and a C-terminal domain (Fig. 2) [28]. An L-shaped hydrophobic tunnel exists inside the AAR molecule. The active center, C294 (based on the residue numbering of AAR from *Synechococcus elongatus* PCC 7942 [*Se*AAR]), is located at the hinge region of the L-shaped tunnel.

The catalytic reaction by AAR occurs via a two-step ping-pong mechanism [28, 29]. In the first step, the substrate acyl-ACP (or acyl-CoA) binds to the first half of the L-shaped tunnel between the N-terminal and NADPH-binding domains. Subsequently, the C1 carbon of the acyl chain forms a thioester bond with C294, thereby releasing ACP (or CoA). In the acyl-enzyme intermediate (Fig. 2), the alkyl group of the acyl chain is located at the second half of the L-shaped tunnel and points toward its exit. In the second step of the catalytic reaction, a coenzyme NADPH binds to an NADPH-binding motif (GXXGXXG; G is glycine and X is any amino acid) in the first half of the L-shaped tunnel. Subsequently, the thioester bond between the acyl chain and C294 is reduced via hydride transfer from NADPH, thereby releasing the aldehyde from the exit of the tunnel.

#### Structure and catalytic mechanism of ADO

**Fig. 3:**
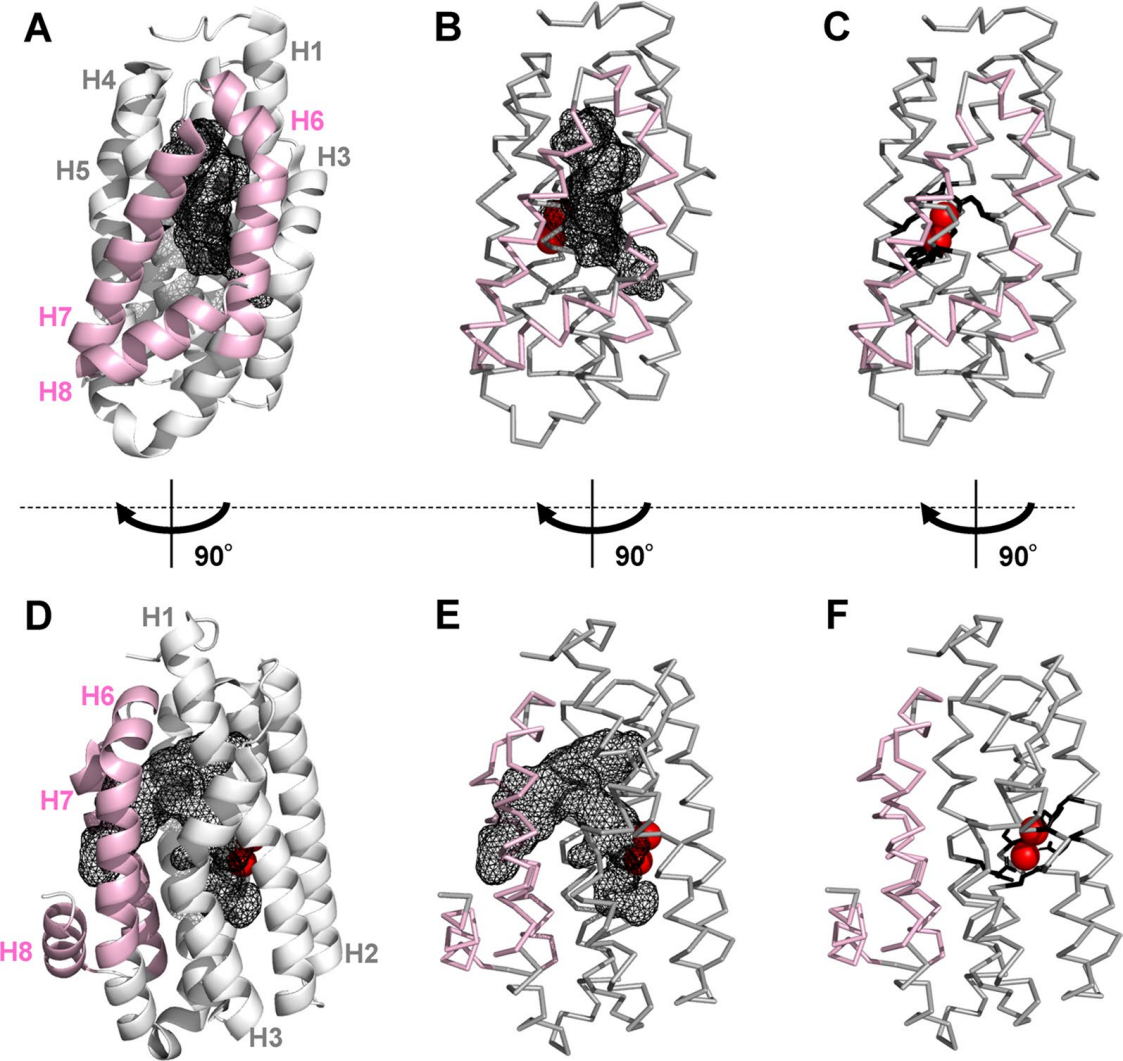
Crystal structure of ADO derived from *Prochlorococcus marinus* MIT 9313 (PDB ID: 4PGI). Structures in (**D**–**F**) are horizontally rotated by 90° from those of (**A**–**C**), respectively. Three α-helices (H6, H7, and H8) comprising the substrate entrance are shown in pink. Two iron atoms are shown as red spheres. In (**A**), (**B**), (**D**), and (**E**), the T-shaped hydrophobic tunnel is shown as a black mesh. In (**B**), (**C**), (**E**), and (**F**), the main chain is shown as wires. In (**C**) and (**F**), the glutamate and histidine residues that chelate two irons are shown as black sticks. ADO, aldehyde deformylating oxygenase

**Fig. 4:**
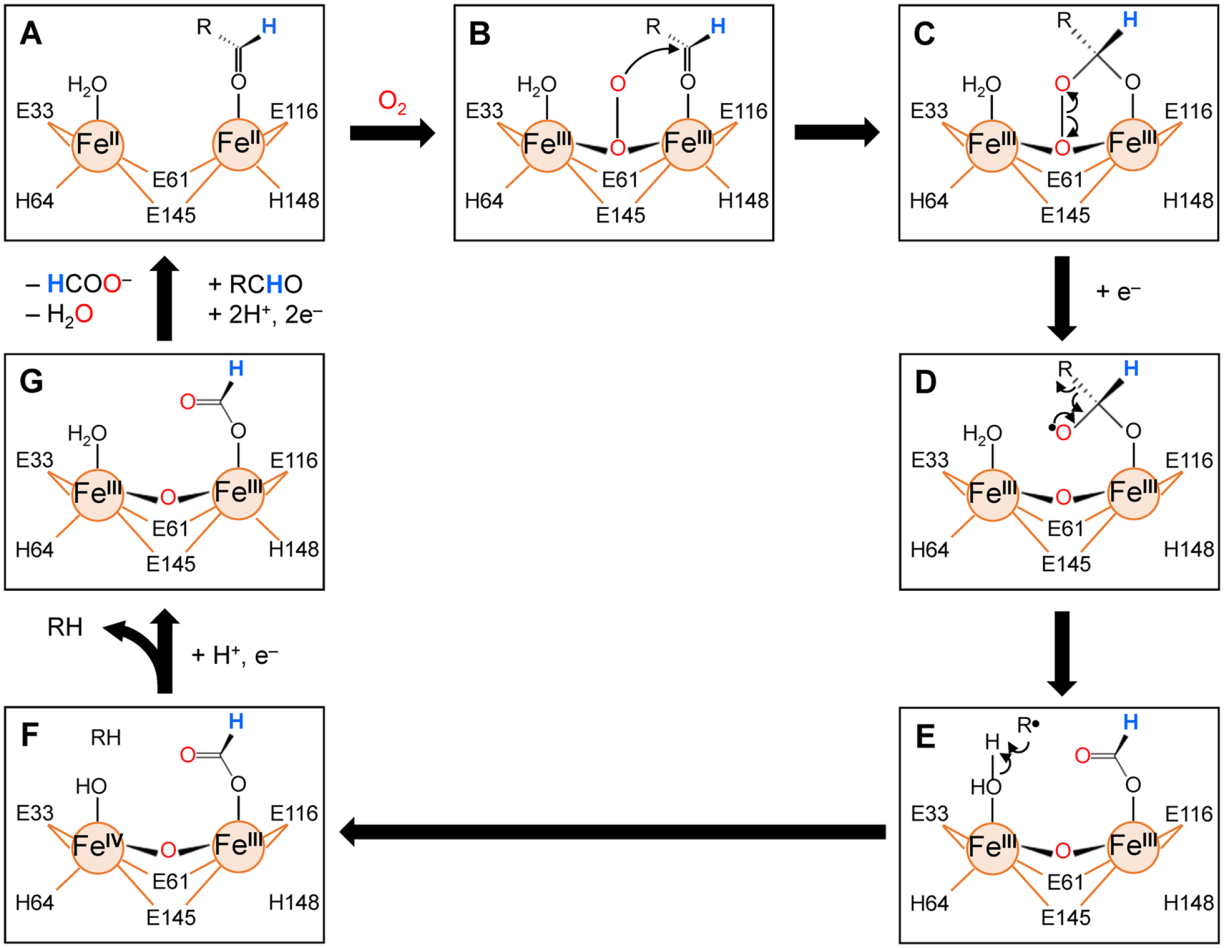
Proposed mechanism of the reaction catalyzed by ADO. The Fe1 (left) and Fe2 (right) iron atoms are shown as orange circles. The coordinate bonds between iron atoms and surrounding residues are shown as orange lines with the residue numbering of ADO derived from *Nostoc punctiforme* PCC 73102. The hydrogen atom derived from aldehyde substrate is shown in blue, and the air-derived oxygen atoms are shown in red. ADO, aldehyde deformylating oxygenase

ADO is a monomeric globular protein consisting of 230–240 amino acid residues. It has an all-α-type structure comprising eight α-helices (H1–H8) (Fig. 3) [28, 30–35]. The entrance of an aldehyde substrate is located at the center of the triangle surrounded by helices H6–H8. From this entrance, the substrate enters a T-shaped tunnel inside the ADO structure. This tunnel is surrounded by hydrophobic residues including tyrosine and phenylalanine on helices H1–H4 and H6, allowing entry of a hydrophobic long-chain aldehyde. At the lower end of the tunnel, two iron atoms forming the active center are bound to two iron-binding motifs (EX_28-29_EX_2_H; E is glutamate and H is histidine) by coordinate bonds (Figs. 3, 4). The first iron atom (Fe1) is coordinated to E33, E61, H64, and E145, while the second iron atom (Fe2) is coordinated to E61, E116, E145, and H148 (based on the residue numbering of ADO from *Nostoc punctiforme* PCC 73102 [*Np*ADO]); E61 and E145 bridge the two iron atoms.

When the aldehyde group of a substrate arrives at the active center, the following catalytic reaction is initiated (Fig. 4) [13, 23, 36–42]: first, an electron lone pair on the oxygen atom of the aldehyde binds to divalent Fe2 (Fig. 4A). Subsequent binding of an air-derived oxygen molecule to both Fe1 and Fe2 makes them trivalent (Fig. 4B), and one of the oxygen atoms nucleophilically attacks the carbonyl of the aldehyde group, forming a peroxyhemiacetal intermediate (Fig. 4C). This intermediate is transformed into a diolyl radical via a one-electron reductive cleavage of the O–O bond (Fig. 4D). The electrons are supplied by a cyanobacterial endogenous reducing system composed of ferredoxin, ferredoxin NADP^+^ reductase, and NADPH [41, 43]. Subsequently, the diolyl radical cleaves the C1–C2 bond of the aldehyde, releasing an alkyl radical with one carbon fewer than that in the substrate aldehyde (Fig. 4E). The alkyl radical acquires a hydrogen atom from a water molecule bound to Fe1, resulting in the formation and release of an alkane (Fig. 4F). Finally, electrons from the reducing system allow the release of a formate from Fe2 (Fig. 4G) and convert trivalent Fe1 and Fe2 into their initial divalent states (Fig. 4A) [24, 44, 45].

### Substrate delivery from AAR to ADO via complex formation

#### Interactions between AAR and ADO

**Fig. 5:**
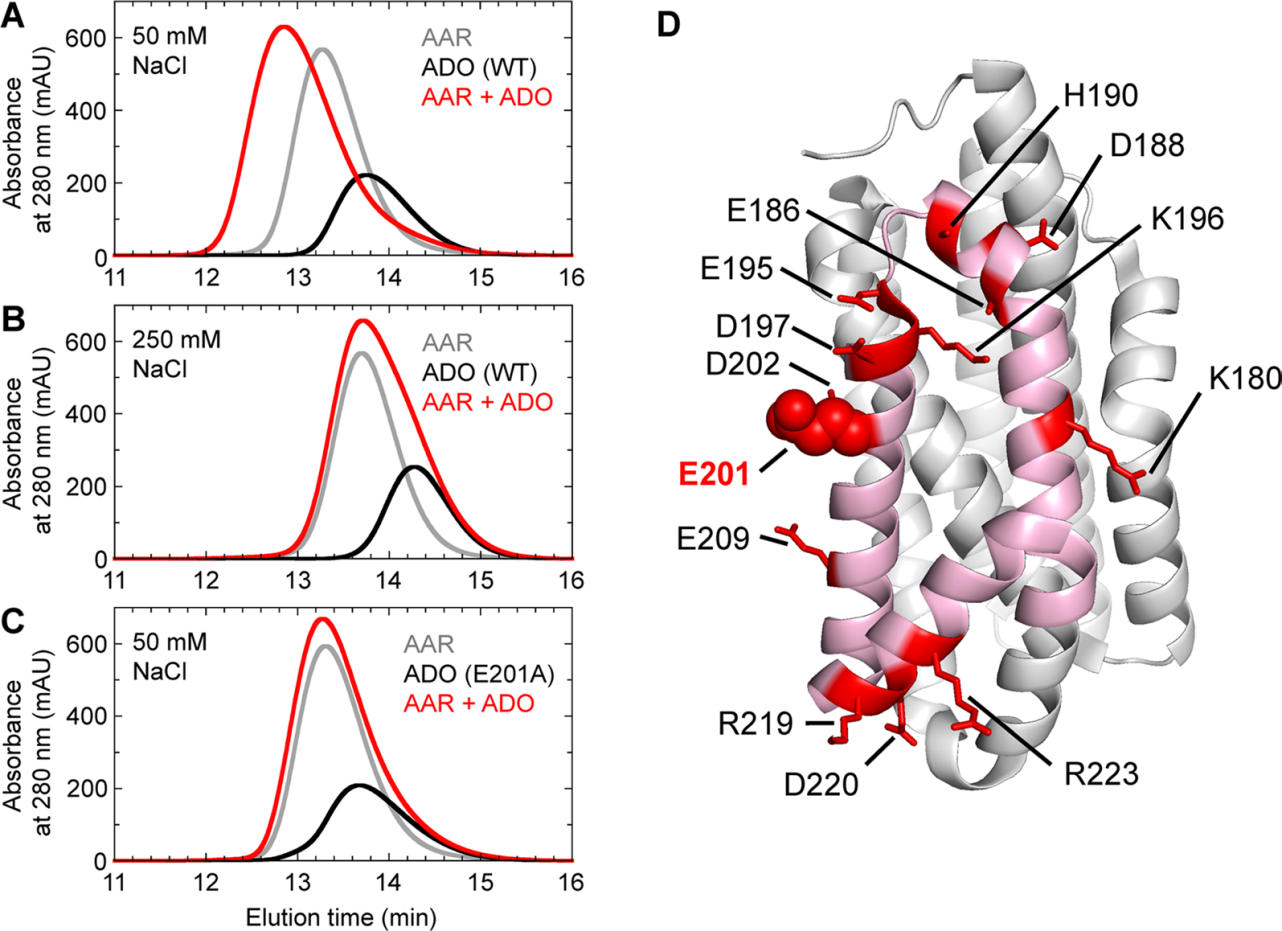
Elution profiles of AAR and ADO measured via size exclusion chromatography [48]. **A–C** Profiles of *Te*AAR only (gray), *Te*ADO only (black), and a mixture of *Te*AAR and *Te*ADO (red). *Te*ADO used was the WT (**A, B**) and E201A mutant (**C**). NaCl concentrations were 50 mM (**A, C**) and 250 mM (**B**). **D** Crystal structure of ADO derived from *Prochlorococcus marinus* str. MIT 9313 (PDB ID: 4TW3). The charged residues on the three α-helices that constitute the substrate entrance (pink) are shown as red sticks, except for E201 (red spheres). Adapted with permission from Ref. [48]. AAR, acyl-(acyl carrier protein) reductase; ADO, aldehyde deformylating oxygenase; *Te*AAR, AAR derived from *Thermosynechococcus elongatus* BP-1; *Te*ADO, ADO derived from *Thermosynechococcus elongatus* BP-1; WT, wild type

The natural substrates of ADO include hexadecanal and octadecanal that are insoluble and form micelles in aqueous solution [46, 47]. Regarding the acquisition of these insoluble substrates by ADO in cells, Warui et al. showed that AAR can efficiently deliver aldehydes to ADO by forming a complex with ADO [47]. Chang et al. investigated the interactions and the binding sites between them in vitro [48]. However, AARs and ADOs derived from mesophilic cyanobacteria (such as *Se*AAR and *Np*ADO) are prone to aggregation [29, 47, 49], preventing in vitro studies of their physical properties. Since proteins derived from thermophilic bacteria generally have high thermal stability and solubility [50], they studied AAR and ADO derived from the thermophilic cyanobacterium *Thermosynechococcus elongatus* BP-1 [51] (*Te*AAR and *Te*ADO, respectively). These proteins are highly soluble and were successfully used in in vitro binding studies. Size-exclusion chromatography revealed that an equal mixture of *Te*AAR and *Te*ADO shows a peak corresponding to the AAR–ADO complex, in addition to the peaks of *Te*AAR and *Te*ADO alone (Fig. 5A) [48]. Moreover, the peak of the complex disappears at high salt concentrations (Fig. 5B), indicating that AAR and ADO bind with each other via electrostatic interactions [48]. If the binding of AAR and ADO were mediated by hydrophobic interactions, the hydrophobic substrates and products would attach to the AAR/ADO-binding sites, thereby inhibiting substrate delivery and product release. Therefore, to efficiently perform an enzymatic reaction consisting of multiple steps, using different interactions at different steps is reasonable: electrostatic interactions for the binding of AAR and ADO and hydrophobic interactions for the binding of proteins and substrates.

#### AAR-binding site on ADO

Alanine scanning mutagenesis was performed on ADO to investigate its AAR-binding sites [48]. Since a triangular region consisting of helices H6–H8 of ADO is considered to be a substrate entrance (Fig. 3) and electrostatic interactions are important for AAR binding, 13 mutants with a single amino acid substitution were generated in which 13 charged residues on helices H6–H8 were replaced with alanine one at a time (Fig. 5D). Among them, the E201A mutant of ADO showed more than 50% reduction in activity [48]. Furthermore, the mutant did not bind to AAR even at low salt concentrations (Fig. 5C). Therefore, the substrate entrance of ADO, composed of helices H6–H8, may represent the interface between AAR and ADO, and E201 is particularly important for the interaction. Later, the crystal structure of the AAR–ADO complex supported these findings [28].

#### Structure of the AAR–ADO complex

**Fig. 6:**
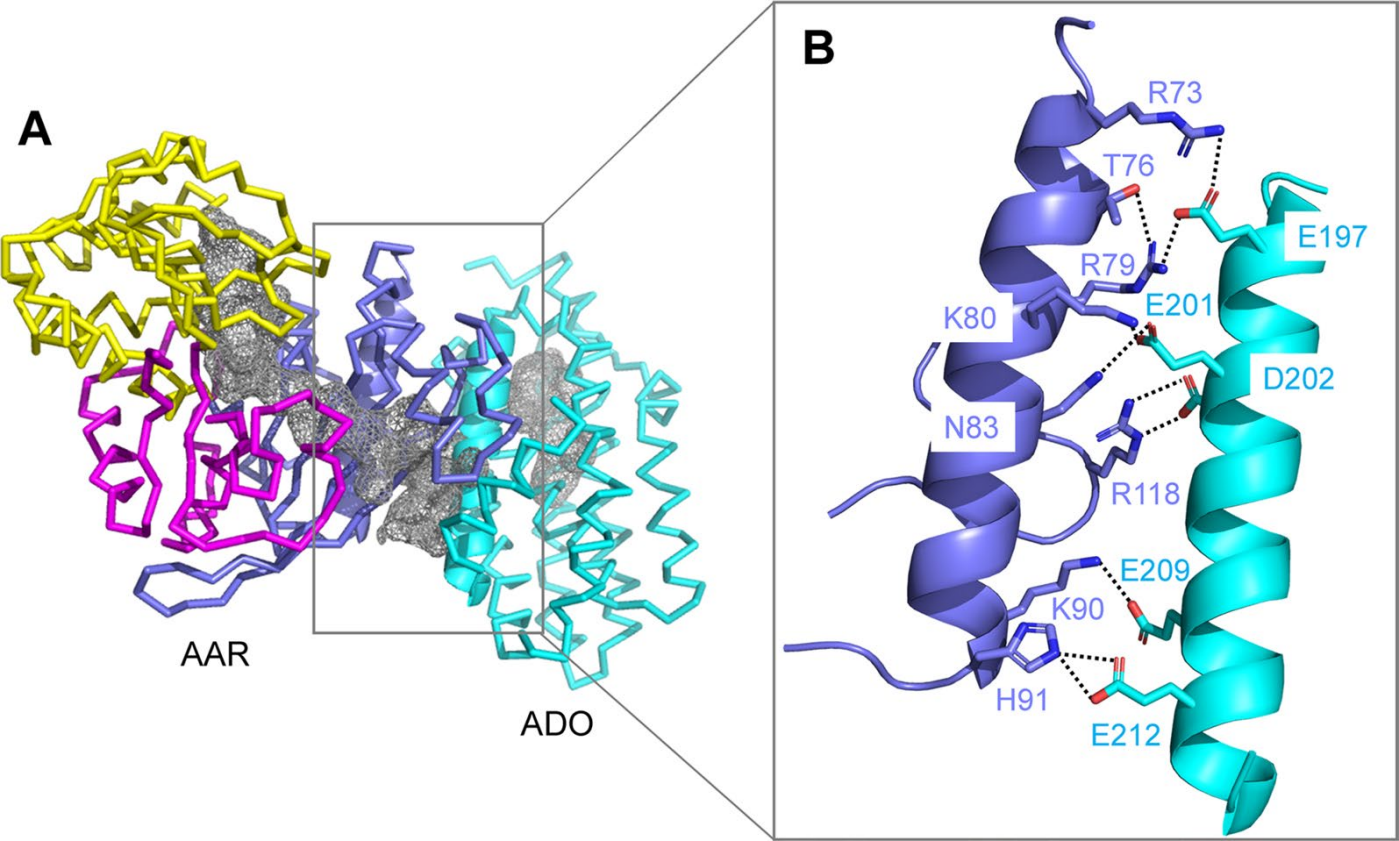
Crystal structure of the *Se*AAR–*Se*ADO complex (PDB ID: 6JZY). **A** The N-terminal, NADPH-binding, and C-terminal domains of AAR are shown in blue, yellow, and magenta, respectively. ADO is shown in light blue. The AAR–ADO binding interface is shown as ribbons. The cavities in AAR and ADO are shown as a gray mesh. **B** Enlarged view of the AAR–ADO binding interface. The interacting residues are shown as sticks, where nitrogen and oxygen atoms are shown in dark blue and red, respectively. The hydrogen bonds and salt bridges are shown as dotted lines. The residue numbers of AAR and ADO are based on the residue numbering of *Se*AAR and ADO derived from *Nostoc punctiforme* PCC 73102, respectively. AAR, acyl-(acyl carrier protein) reductase; ADO, aldehyde deformylating oxygenase; *Se*AAR, AAR derived from *Synechococcus elongatus* PCC 7942; *Se*ADO, ADO derived from *Synechococcus elongatus* PCC 7942

The crystal structure of the AAR–ADO complex shows that the conserved acidic residues on helix H7 of ADO (E197, E201, and D202; based on the residue numbering of *Np*ADO) form salt bridges with the conserved basic residues at the N-terminal domain of AAR (R73, R79, K80, and R118) via electrostatic interactions (Fig. 6) [28]. Moreover, substitution of these residues with alanine prevents the binding of both proteins, demonstrating that these electrostatic interactions are essential for complex formation [28]. Notably, in the AAR–ADO complex structure, the exit of the L-shaped hydrophobic tunnel of AAR is close to the entrance of the T-shaped hydrophobic tunnel of ADO. Therefore, hydrophobic long-chain aldehydes synthesized by AAR are directly delivered to ADO in an efficient manner without external release.

### Alkane production in microorganisms using AAR and ADO

The discovery of AAR and ADO enabled the production of alkanes in genetically engineered microorganisms [13, 52]. Schirmer et al. succeeded in producing 300 mg/L of alkanes/alkenes by co-expressing *Se*AAR and *Np*ADO in *E. coli* [22]. Wang et al. succeeded in increasing photosynthetic production of alkanes in *Synechocystis* sp. PCC 6803 from 300 to 700 μg/L/OD by overexpression of AAR and ADO from cyanobacterial species [53]. Furthermore, Yoshida et al. overexpressed AAR and ADO from *Synechocystis* sp. PCC 6803 (6803AAR and 6803ADO, respectively) in *Synechocystis* sp. PCC 6803 and *Limnothrix/Pseudanabaena* sp. strain ABRG5-3, which undergoes autolysis by changing culture conditions, and successfully produced heptadecane up to 50–60% of the dry cell weight [54, 55]. Alkane production via overexpression of ADO has also been reported in organisms other than *E. coli* and cyanobacteria, including yeast [56–59], *Clostridium thermocellum* [60], *Cupriavidus necator* [61], *Aspergillus carbonarius* ITEM 5010 [62], *Acinetobacter baylyi* ADP1 [63], and *Rhodococcus opacus* [64].

Metabolic engineering has been used in many studies to improve the efficiency of alkane production. In particular, studies have focused on the bacterial type-I and type-II fatty acid synthesis pathways, in which acyl-ACPs, the substrates of AARs, are produced during the processes [65–69]. In type-II fatty acid synthesis pathway of *E. coli*, excess levels of acyl-ACPs in cells cause feedback inhibition, thereby suppressing fatty acid synthesis. To enable the overproduction of acyl-ACPs, Coursolle et al. introduced a type-I fatty acid synthesis pathway together with AAR and ADO into *E. coli* and successfully produced 57 mg/L of pentadecane [70]. In addition, Song et al. reported that both deletion of alcohol dehydrogenase YqhD, which converts aldehydes to alcohols, and expression of a transcription factor FadR, which activates fatty acid synthesis, dramatically increased alkane production from 24 mg/L to 256 mg/L by co-expression of AAR and ADO in *E. coli* [71].

The aforementioned studies used AARs and ADOs derived from cyanobacteria by synthetic biology and metabolic engineering approaches. However, which AAR and ADO have the highest activity among those derived from various cyanobacteria has been unclear. Identifying AARs and ADOs that show high activities and increasing their activities using protein engineering may improve the efficiency of alkane production in microorganisms.

### Improvement of AAR activity

#### Identifying AARs that show high activity

Various cyanobacteria possess AAR genes. Kudo et al. compared the activity of AARs derived from 12 representative cyanobacteria (Table 1) [72]. Since AARs tend to aggregate [29, 47, 49], a method to measure AAR activity using *E. coli* was developed [72]. Here, *E. coli* co-expressing *AAR* and *ADO* genes are cultured under conditions where the enzymatic reaction of AAR is the rate-limiting step in alkane synthesis. After sonication of the cell cultures, alkanes/alkenes produced within cells are extracted using ethyl acetate and quantified via gas chromatography–mass spectrometry (GC–MS). The amount of AAR present in the soluble fraction of *E. coli* is quantified via western blotting. Subsequently, the total amount of the produced alkanes/alkenes is divided by the amount of soluble AAR to determine AAR activity [72].

**Table 1:** AARs derived from 12 representative cyanobacteria used for activity measurements

Abbreviation	Derived cyanobacterial species
*Freshwater cyanobacteria*	
* Se*AAR	*Synechococcus elongatus* PCC 7942
* Gv*AAR	*Gloeobacter violaceus* PCC 7421
* Np*AAR	*Nostoc punctiforme* PCC 73102
* Te*AAR	*Thermosynechococcus elongatus* BP-1
* Ma*AAR	*Microcystis* *aeruginosa*
6803AAR	*Synechocystis* sp. PCC 6803
*Marine cyanobacteria*	
* Pm*AAR	*Prochlorococcus marinus* str. MIT 9313
7336AAR	*Synechococcus* sp. PCC 7336
9917AAR	*Synechococcus* sp. RS9917
0205AAR	*Synechococcus* sp. CB0205
1986AAR	*Prochlorococcus marinus* subsp. pastoris str. CCMP1986
51142AAR	*Cyanothece* sp. ATCC 51142

AARs derived from freshwater or marine cyanobacteria are listed in the order of decreasing activity

*AAR* acyl-(acyl carrier protein) reductase

**Fig. 7:**
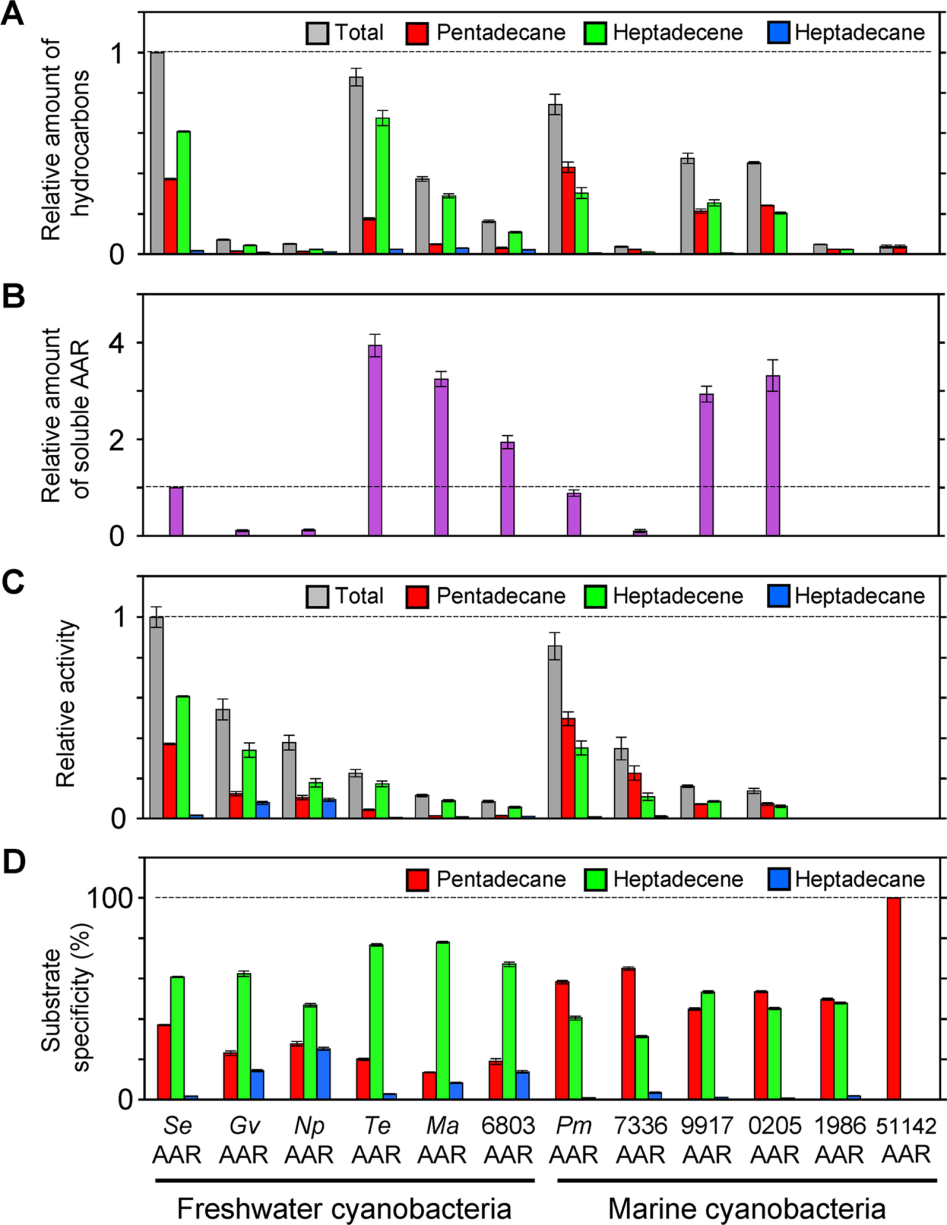
Activity and substrate specificity of AARs derived from 12 representative cyanobacteria (Table 1) [72]. **A** The relative amount of total hydrocarbons (gray), pentadecane (red), heptadecene (green), and heptadecane (blue) produced in *Escherichia coli* co-expressing genes encoding aldehyde deformylating oxygenase from *Nostoc punctiforme* PCC 73102 and the indicated AAR. The values are normalized to the total amount of hydrocarbons produced when *Se*AAR was used. **B** Amount of soluble AAR relative to that of *Se*AAR. **C** Activity of AAR relative to that of *Se*AAR. **D** Fractions of pentadecane, heptadecene, and heptadecane relative to the total amount of hydrocarbons produced in *E. coli*, indicating the substrate specificity of AAR. AAR, acyl-(acyl carrier protein) reductase; *Se*AAR, AAR from *Synechococcus elongatus* PCC 7942

Kudo et al. found that AARs derived from various cyanobacterial species have different activities [72]. When co-expressed with *Np*ADO in *E. coli*, the total amount of alkanes/alkenes produced was highest for *Se*AAR, followed by that for *Te*AAR and AAR derived from *Prochlorococcus marinus* MIT 9313 (*Pm*AAR) (Fig. 7A). A large difference was also observed in the amount of soluble AAR in *E. coli* for different AARs. *Te*AAR showed the highest amount, while *Se*AAR and *Pm*AAR showed low amounts (Fig. 7B). Finally, the AAR activity was highest for *Se*AAR, followed by *Pm*AAR and AAR derived from *Gloeobacter violaceus* PCC 7421 (*Gv*AAR), while the activity was low for 6803AAR, *Te*AAR, and AAR derived from *Synechococcus* sp. PCC 7336 (7336AAR) (Fig. 7C). Therefore, *Se*AAR may facilitate efficient alkane production in microorganisms, if one of the wild-type AARs is used for heterologous expression.

#### Substrate specificity of AARs

The aforementioned studies showed that substrate specificity differs among various AARs [72]. *E. coli* cells co-expressing *AAR* and *ADO* mainly produce pentadecane (15 carbons) and heptadecene (17 carbons). Notably, the proportions of pentadecane and heptadecene produced differ for AARs derived from different species (Fig. 7D). Therefore, AARs may have different substrate specificities, resulting in the production of different aldehydes. Comparison of the produced aldehydes with a molecular phylogenetic tree based on the amino acid sequences of AAR revealed that AARs from marine cyanobacteria (such as *Pm*AAR and 7336AAR) mainly produce 16-carbon aldehydes, while AARs from freshwater cyanobacteria (such as *Se*AAR, *Te*AAR, and *Gv*AAR) produce 18-carbon aldehydes (Fig. 7D). These results suggest that the substrate specificity of AARs has evolved according to the growth environment of cyanobacterial species [72]. In contrast, ADO has low substrate specificity and can use various aldehydes as substrates [73]. Therefore, selecting an appropriate AAR is important for the selective production of alkanes/alkanes with different lengths of the carbon chain. For example, for production of diesel oils with low freezing points for use in cold regions, AARs that produce aldehydes with short carbon lengths can be used, such as the highly active 7336AAR mutants described later [74].

#### Engineering of AAR to improve hydrocarbon production

**Fig. 8:**
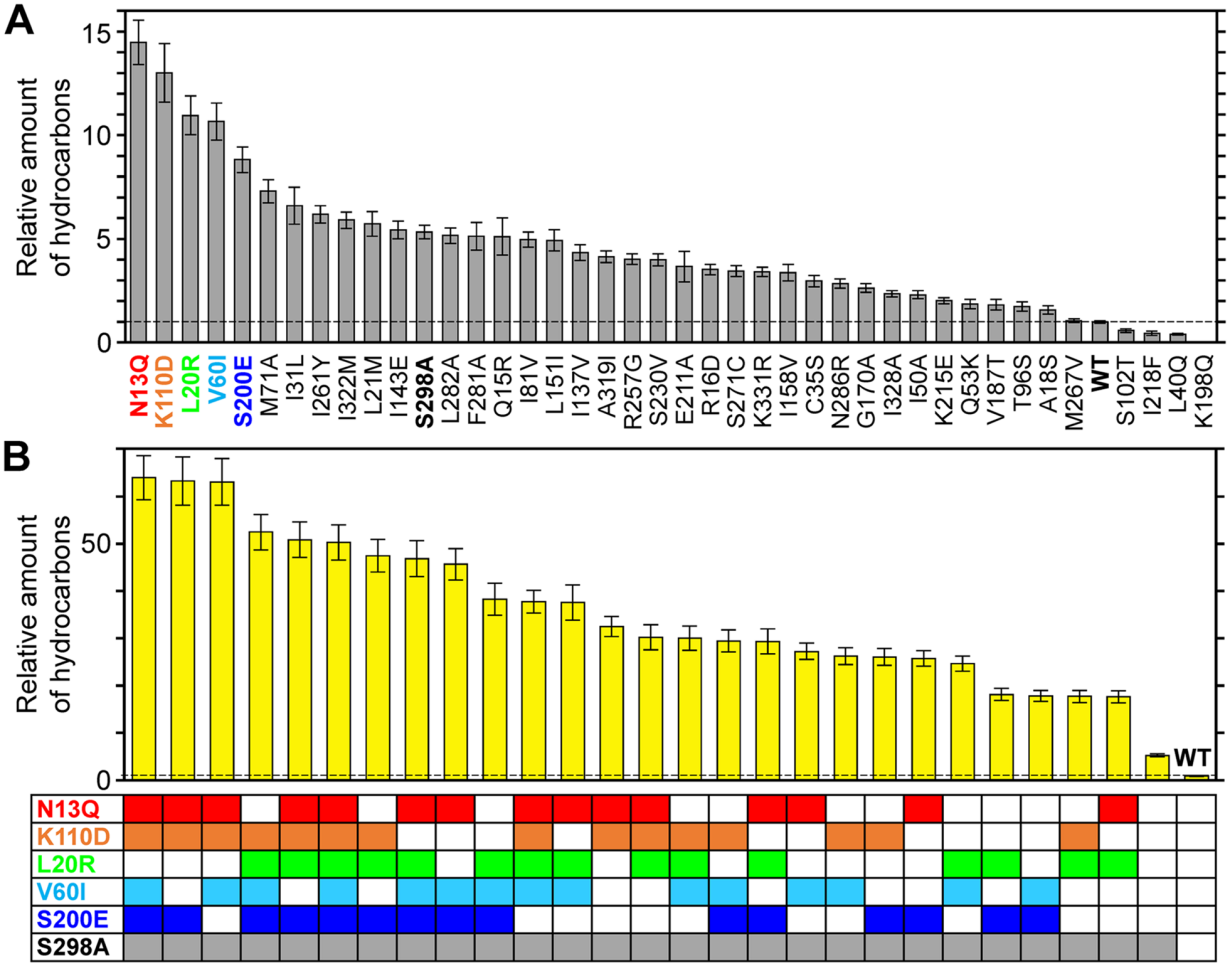
Amount of total hydrocarbons produced in *Escherichia coli* co-expressing *Np*ADO and the indicated 7336AAR mutant [74]. The values were normalized with respect to that of the WT 7336AAR. **A** The 40 mutants of 7336AAR with a single amino acid substitution using the S298A mutant as a pseudo-WT. **B** The 26 mutants of 7336AAR having a combination of up to six substitutions shown as colored boxes at the bottom of the panel. In (**A**) and (**B**), the results for the WT and the S298A mutant are also shown. AAR, acyl-(acyl carrier protein) reductase; *Np*ADO, aldehyde deformylating oxygenase from *Nostoc punctiforme* PCC 73102; WT, wild type

Although the turnover number of AAR is higher than that of ADO, it is significantly lower than that of most enzymes [22]. Therefore, for use of AAR and ADO in bioalkane production, increasing AAR activity is necessary by introducing amino acid substitutions. Since evolutionarily conserved amino acid residues are generally crucial for structure and function of proteins, enzymatic activity may be improved via amino acid substitutions at non-conserved residues. To search for such mutation sites, Kudo et al. introduced amino acids found in highly active AARs, including *Se*AAR and *Pm*AAR, into a less active AAR (7336AAR) at 41 non-conserved sites and examined whether the mutations enhanced 7336AAR activity [74]. The activity of the wild-type 7336AAR is difficult to measure owing to its low expression in *E. coli*; however, S298A substitution at one of the non-conserved sites greatly increases protein expression [74]. Therefore, single amino acid substitutions were introduced at 40 non-conserved sites of 7336AAR using the S298A mutant as a pseudo-wild type. Among them, six amino acid substitutions enhance hydrocarbon production by improving either the activity or the amount of soluble AAR (Fig. 8) [74]. Next, 26 multiple mutants were created with various combinations of the six amino acid substitutions. Among them, three multiple mutants successfully increase hydrocarbon production by more than 60-fold compared with that of the wild-type 7336AAR (Fig. 8) [74]. Remarkably, the amount of hydrocarbons produced by these mutants was 1.5-fold higher than that of the most active AAR (*Se*AAR), indicating that these mutants are most effective in producing hydrocarbons, particularly pentadecane, among various AARs reported so far.

### Improvement of ADO activity

#### Identifying ADOs that show high activity

**Fig. 9:**
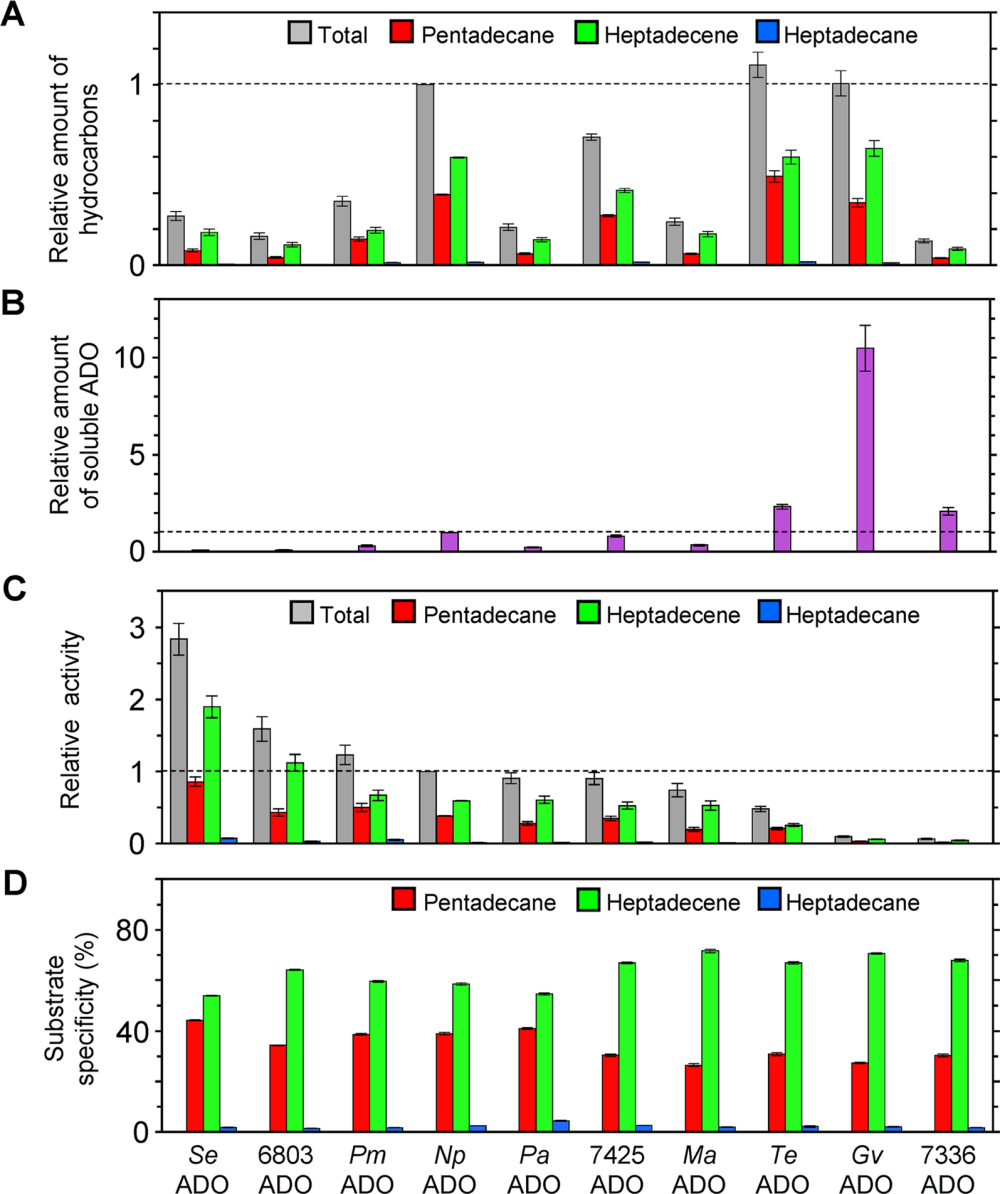
Activity and substrate specificity of ADOs derived from 10 representative cyanobacteria (Table 2) [73]. **A** The relative amount of total hydrocarbons (gray), pentadecane (red), heptadecene (green), and heptadecane (blue) produced in *Escherichia coli* co-expressing genes encoding acyl-(acyl carrier protein) reductase derived from *Synechococcus elongatus* PCC 7942 and the indicated ADO. The values were normalized with respect to the total amount of hydrocarbons produced when *Np*ADO was used. **B** The amount of soluble ADO relative to that of *Np*ADO. **C** Activity of ADO relative to that of *Np*ADO. **D** Fractions of pentadecane, heptadecene, and heptadecane relative to the total amount of hydrocarbons produced in *E. coli*, indicating the substrate specificity of ADO. ADO, aldehyde deformylating oxygenase; *Np*ADO, ADO derived from *Nostoc punctiforme* PCC 73102

ADOs have a low turnover number that is at most 1 min^−1^ [45]. Therefore, for facilitating practical application of ADO in bioalkane production, searching for ADOs showing high activity among those derived from various cyanobacteria and designing mutant ADOs for increased activity are necessary. As mentioned earlier, the substrates of ADO, 16–18 carbon aldehydes, are insoluble and form micelles in aqueous solution. This prevents an accurate determination of ADO activity in vitro because the dissociation of an aldehyde molecule from micelles represents the rate-limiting step of the overall catalytic reaction [44, 46]. Since ADOs can efficiently receive substrates from AARs in cells, Kudo et al. performed co-expression of *Se*AAR showing high activity with one of the ADOs derived from 10 representative cyanobacteria (Table 2) in *E. coli* cells, cultured them under conditions where ADO reaction is the rate-limiting step, and quantified the produced alkanes/alkenes via GC–MS [73]. ADO activity was determined by dividing the amount of total hydrocarbon produced by the amount of soluble ADO protein quantified via sodium dodecyl sulfate—polyacrylamide gel electrophoresis or western blotting [73, 75]. They found that the proportions of pentadecane, heptadecene, and heptadecane produced were almost the same for all ADOs (Fig. 9D). This indicates that substrate specificity is similar for ADOs derived from various cyanobacteria. In contrast, total alkane production varies widely among ADOs (Fig. 9A): highest for *Te*ADO, followed by *Gv*ADO and *Np*ADO. In addition, the amount of soluble ADO is high for *Gv*ADO and *Te*ADO (Fig. 9B). Consequently, the activities of *Gv*ADO and *Te*ADO are low (Fig. 9C). Notably, the activity of *Se*ADO is highest, followed by those of 6803ADO, *Pm*ADO, and *Np*ADO, although previous studies have suggested that *Np*ADO shows the highest activity [22]. *Te*ADO is derived from a thermophilic cyanobacterium with an optimum growth temperature of 55 °C [51], suggesting that *Te*ADO shows high activity at high temperatures.

**Table 2:** ADOs derived from 10 representative cyanobacteria used for activity measurements

Abbreviation	Derived cyanobacterial species
*Freshwater cyanobacteria*	
* Se*ADO	*Synechococcus elongatus* PCC 7942
6803ADO	*Synechocystis* sp. PCC 6803
* Np*ADO	*Nostoc punctiforme* PCC 73102
* Pa*ADO	*Planktothrix agardhii* NIVA-CYA 126/8
7425ADO	*Cyanothece* sp. PCC 7425
* Ma*ADO	*Microcystis aeruginosa*
* Te*ADO	*Thermosynechococcus elongatus* BP-1
* Gv*ADO	*Gloeobacter violaceus* PCC 7421
*Marine cyanobacteria*	
* Pm*ADO	*Prochlorococcus marinus* str. MIT 9313
7336ADO	*Synechococcus* sp. PCC 7336

ADOs derived from freshwater or marine cyanobacteria are listed in the order of decreasing activity

*ADO* aldehyde deformylating oxygenase

#### Engineering of ADO to improve hydrocarbon production

**Fig. 10:**
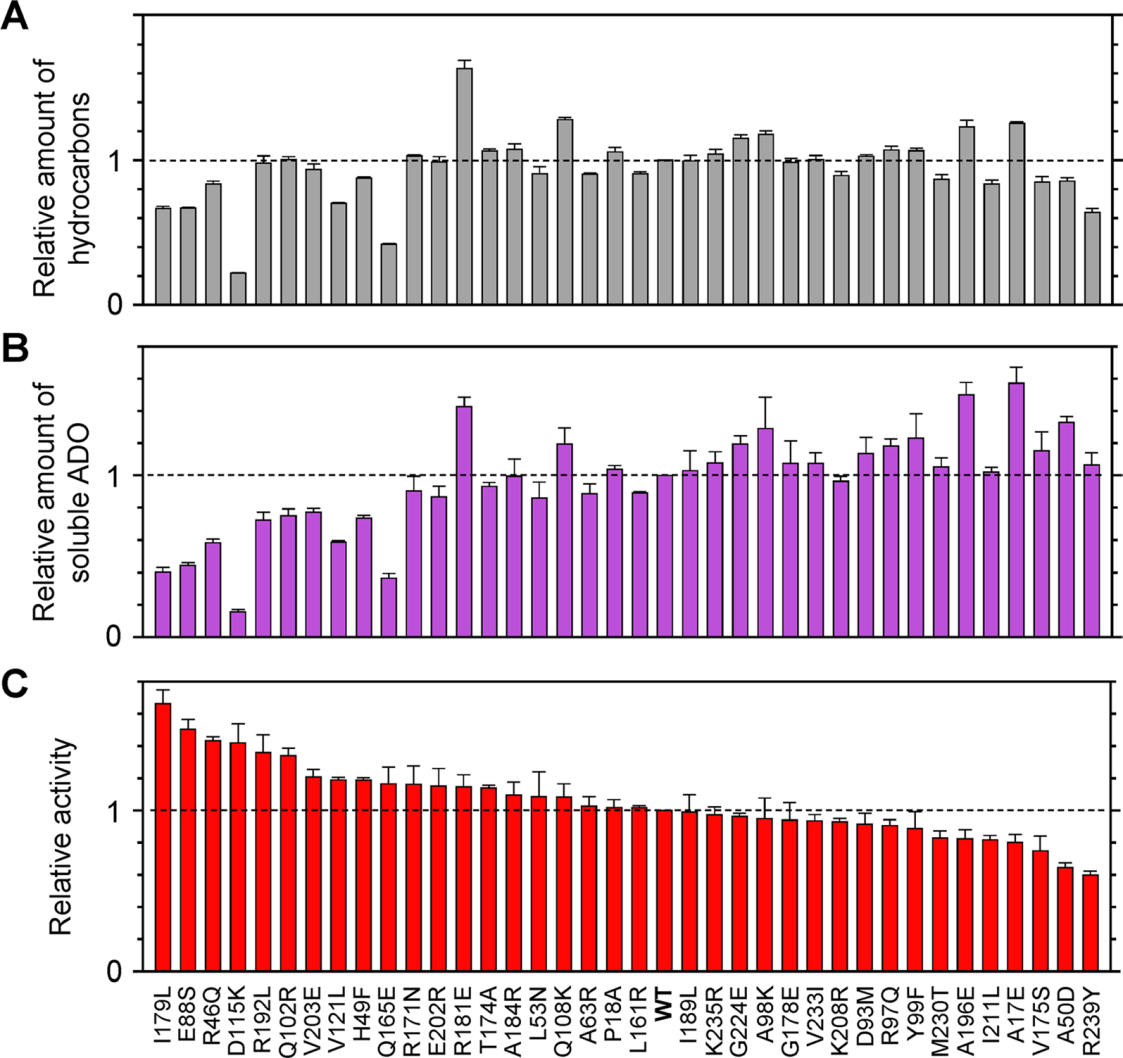
Activity of 37 mutants of *Gv*ADO with a single amino acid substitution [73]. **A** The amount of total hydrocarbons produced in *Escherichia coli* cells co-expressing genes encoding acyl-(acyl carrier protein) reductase derived from *Synechococcus elongatus* PCC 7942 and the indicated mutant of *Gv*ADO. The values were normalized with respect to that of the WT *Gv*ADO. **B** The amount of soluble ADO of the mutants relative to that of WT. **C** Activity of the mutants relative to that of WT. ADO, aldehyde deformylating oxygenase; *Gv*ADO, ADO derived from *Gloeobacter violaceus* PCC 7421; WT wild type

The difference in ADO activity must be owing to the differences in amino acid residues at non-conserved sites. To identify residues that are important in determining ADO activity, Kudo et al. created 37 single ADO mutants in which 37 non-conserved residues of ADO showing low activity (*Gv*ADO) were replaced by those of an ADO showing high activity (*Se*ADO) one at a time (Fig. 10). Among them, 20 mutants of *Gv*ADO showed increased activity compared with that of the wild type; therefore, 20 amino acid residues were identified that determine ADO activity. I179L showed the highest activity with a 60% increase compared to that of wild-type *Gv*ADO (Fig. 10C). In addition, three mutants (E88S, R46Q, and D115K) and six other mutants (R192L, Q102R, V203E, V121L, H49F, and Q165E) showed increases in activities by more than 40% and more than 17%, respectively.

Increasing both the activity and amount of soluble ADO is important to improve the efficiency of alkane production using microorganisms. Although the aforementioned 10 mutants showed increased activity by more than 17% compared to that of the wild type, the amount of soluble ADO decreased (Fig. 10B). In contrast, the remaining 10 out of the 20 mutants with activities higher than the wild type maintained more than 85% of the amount of soluble ADO (Fig. 10B). Moreover, among all 37 mutants, three (A17E, R181E, and A196E) showed increased amount of soluble ADO by more than 40% as well as the total amount of alkanes produced (Fig. 10A, B). In particular, R181E showed the highest amount of total alkane production; it presented 15% higher activity and 43% higher amount of soluble ADO than did the wild type, resulting in a 60% increase in total alkane production.

#### Amino acid residues important for improving ADO activity

**Fig. 11:**
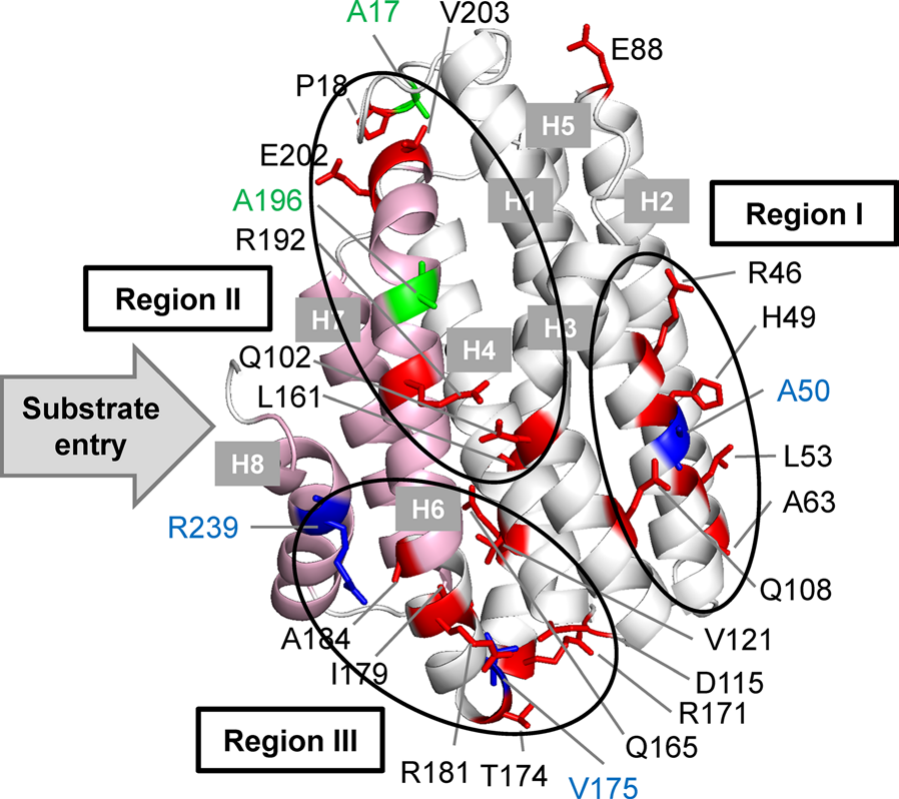
Mapping the mutation sites onto *Gv*ADO structure predicted by homology modeling [73]. Mutations at 20 sites increased ADO activity (red), those at three sites decreased activity by more than 20% (blue), and those at two sites increased the amount of soluble ADO by more than 50% (green sticks). Mutation sites are classified into the three regions indicated by black circles. The three α-helices that constitute the substrate entrance are highlighted in pink. The direction of substrate entry is shown using a gray arrow. ADO, aldehyde deformylating oxygenase; *Gv*ADO, ADO derived from *Gloeobacter violaceus* PCC 7421

The mutation sites that had considerable effects on ADO activity can be classified into Regions I, II, and III (Fig. 11) [73].

Region I (R46, H49, A50, L53, A63, and Q108) contains the active center (iron atoms) of ADO (Fig. 11). Amino acid substitutions in this region increase the activity by potentially perturbing the active center.

Region II (A17, P18, Q102, L161, R192, A196, E202, and V203) is adjacent to the substrate entrance (that is, product exit) of ADO and contains the AAR-binding site (Fig. 11). Furthermore, this region is involved in binding to ferredoxin [76]. Amino acid substitutions in region II possibly enhance ADO activity by perturbing substrate entry, product release, AAR binding, and/or ferredoxin binding. Since A17 and A196 are present on the surface of ADO, substitution of hydrophobic residues (alanine) with hydrophilic residues (glutamate) in the A17E and A196E mutations may stabilize the structure, thereby increasing the amount of soluble ADO by more than 50%.

Region III (D115, V121, Q165, R171, T174, V175, I179, R181, A184, and R239) is located near the edge of the long α-helices that constitute the substrate entrance (Fig. 11). This region may function as a hinge to control the open/close dynamics of the substrate entrance in Region II, thereby affecting its activity.

In addition to the above consideration, the mutations may affect ADO stability, leading to changes in activity and/or amount of soluble fraction.

#### Degradation sequence at the C-terminus of ADO

Alkane biosynthesis using ADO is affected by the low ADO expression (or low accumulation) in vivo [73, 77]. To search for ADO mutants with high expression, Liu et al. performed directed evolution experiments and found that mutations at the C-terminal helix H8 or its removal increased ADO levels in *E. coli* [76]. They identified a degradation sequence (called “degron”; the consensus sequence is RMSAYGLAAA) at the C-terminus of ADO, which promotes protein degradation via the ATP-dependent proteases ClpAP and Lon [76]. *Gv*ADO, which is highly expressed in *E. coli* (Fig. 9B), does not contain this degron sequence. Moreover, deletion of the C-terminal degron from ADO increases its accumulation in cells but simultaneously reduces its activity [76]. Therefore, to improve the efficiency of alkane biosynthesis via microbial expression of ADO, the development of ADO mutants with modified C-terminal degron sequence to increase the intracellular accumulation of ADO without decreasing its activity is necessary.

### Role of alkanes in cyanobacteria

As described above, *Se*AAR and *Se*ADO showing high activity have been identified among various cyanobacterial AARs and ADOs, respectively, and their mutants showing high activity have also been developed [72–74]. Introducing these AARs and ADOs into cyanobacteria will enable efficient carbon—neutral production of alkanes. Because overproduction of alkanes/alkenes may affect the growth of cyanobacteria, understanding the role of alkanes in cyanobacteria is important. Hydrocarbons primarily serve as barriers to water in higher plants and as pheromones in insects [9, 78]. In contrast, the role of alkane production in cyanobacteria has been unknown, although most cyanobacteria harbor genes for alkane production [22, 79].

Lea-Smith et al. constructed mutant strains of *Synechocystis* sp. PCC 6803 and *Synechococcus* sp. PCC 7002 lacking *AAR*/*ADO* and the olefin synthase pathway, respectively [80]. These hydrocarbon-deficient strains show reduced growth, enlarged cell size, and increased defects in cell division. Therefore, alkanes may provide flexibility to membranes, which is necessary for optimizing cell division, size, and growth [80, 81].

Alkanes synthesized in cyanobacteria have been suggested to serve as electron sinks [79]. Berla et al. reported that a mutant of *Synechocystis* sp. PCC 6803 lacking *AAR/ADO* exhibits poor growth at low temperatures and enhanced cyclic electron flow [82], which regulates the redox state of cells by suppressing reactive oxygen species (ROS) generation [83]. The authors argue that the cyclic electron flow is enhanced in alkane-deficient cyanobacteria to maintain the redox balance, suggesting that alkanes are critical metabolites for maintaining the redox balance during photosynthesis [82]. Similarly, a mutant strain of *Synechococcus elongatus* PCC 7942 lacking *AAR/ADO* shows growth defects under high salt concentrations, whereas heterologous expression of AAR and ADO derived from halotolerant *Aphanothece halophytica* restores growth of the *Synechococcus* mutant at high salinity [84]. Since cyanobacteria produce ROS under high salinity stress [85], these results indicate that alkanes maintain redox balance in cyanobacteria.

Notably, Qiao et al. recently reported that under high-light conditions, ADO converts alkanes in cell membrane to alcohols or aldehydes, which are then converted into fatty acids by aldehyde dehydrogenase to maintain lipid homeostasis [86]. Since alkanes can serve as electron donors to further reduce partially reduced ROS, the authors proposed that alkane degradation by ADO represents an emergency mechanism for responding to oxidative stress induced by high-light conditions. This suggests that high-light irradiation should be avoided to increase the efficiency of alkane biosynthesis in cyanobacteria [86].

These studies indicate that alkanes play important roles in the growth and redox maintenance of cyanobacteria. Further studies should clarify the effects of alkane overproduction on cyanobacteria, to improve cyanobacterial production of alkanes.

## Conclusion and perspectives

This review summarizes previous findings on the structure and function of AAR and ADO, which are essential for alkane synthesis in cyanobacteria, and recent efforts to improve the activities of AAR and ADO for efficient bioalkane production. Structural and mutational analyses of AAR and ADO have contributed to elucidating the mechanisms of their functions. Recent studies have also revealed that AAR and ADO form a binary complex via electrostatic interactions and efficiently deliver aldehydes from AAR to ADO. Furthermore, overexpression of AAR and ADO in genetically engineered microorganisms such as *E. coli* and yeasts has enabled the production of various alkanes, including short-, long-, and branched-chain alkanes. However, both enzymes have low activity; in particular, the reaction catalyzed by ADO represents a bottleneck in bioalkane production. Therefore, protein engineering studies have been conducted to improve the activity of AAR and ADO, and mutants showing high activity of these enzymes have been generated. Introducing these mutated enzymes into cyanobacteria is expected to facilitate carbon—neutral production of biofuels. In addition, to increase alkane production in cyanobacteria, the role of alkanes in cyanobacteria needs to be understood. Recent studies have suggested that alkanes may protect the photosynthetic system from oxidative stress produced in response to external stresses such as temperature and light. Note that accurate determination of the kinetic parameters (turnover number *k*_cat_ and Michaelis constant *K*_m_) of AAR and ADO is challenging due to the high aggregation propensity of the AAR proteins and the low solubility of the natural substrates of ADO; however, it is important to obtain these parameters for detailed understanding of the catalytic properties of AAR and ADO.

Considerable progress has been made in recent studies on AAR and ADO. However, further improvements of these enzymes are necessary to enable their use in industrial production of diesel oils. Experimental approaches, such as directed evolution [87], and theoretical approaches of protein design, such as the use of Rosetta software [88] and the state-of-the-art deep learning methods [89, 90], will be useful for further increasing the activity and expression of AAR and ADO in microorganisms. The combination of improved mutants of *AAR/ADO* with various metabolic engineering approaches to overexpress them and increase substrate supply in microorganisms will enable efficient production of bioalkanes. Formate, which is a byproduct of alkane production by ADO, has recently attracted attention as a hydrogen carrier in fuel cells [91, 92]. Therefore, improving alkane production by AAR and ADO in cyanobacteria will enable carbon—neutral production of sustainable bioenergy, which is in line with the Sustainable Development Goals, thereby reducing global warming.

## Data Availability

Not applicable.

## Abbreviations

AAR: Acyl-ACP reductase
ADO: Aldehyde-deformylating oxygenase
ACP: Acyl carrier protein
CoA: Coenzyme A
NADPH: Nicotinamide adenine dinucleotide phosphate
GC–MS: Gas chromatography–mass spectrometry
ROS: Reactive oxygen species
